# Supplementary material for: Enhanced Surface Accessibility of SARS-CoV-2 Omicron Spike Protein Due to an Altered Glycosylation Profile
Source: ACS Infect Dis. 2024 May 10;10(6):2032–46. doi: 10.1021/acsinfecdis.4c00015 (PMC11184558; doi:10.1021/acsinfecdis.4c00015)
Supplement: Supplementary file 1 — id4c00015_si_001.pdf [file id4c00015_si_001.pdf]

Supplementary Materials for

Enhanced surface accessibility of SARS-CoV-2 Omicron spike protein due to an altered glycosylation profile

Dongxia Wang[1†]*, Zijian Zhang[2†], Jakub Baudys[1], Christopher Haynes[1], Sarah H. Osman[1], Bin Zhou[3], John R. Barr[1], James C. Gumbart[2]*

[1]National Center for Environmental Health, Division of Laboratory Sciences. Centers for Disease Control and Prevention (CDC), Atlanta, Georgia, 30322 USA.

[2]School of Physics, Georgia Institute of Technology, Atlanta, Georgia, 30332 USA.

[3]National Center for Immunization and Respiratory Diseases, Centers for Disease Control and Prevention (CDC), Atlanta, Georgia, 30322 USA.

†These authors made equal contribution to this work

*To whom correspondence should be addressed.

Dongxia Wang, Ph.D.: telephone 770-488-0446, email dov2@cdc.gov, fax 770-488-0509

James C. Gumbart, Ph.D.: telephone 404-385-0797, email gumbart@physics.gatech.edu

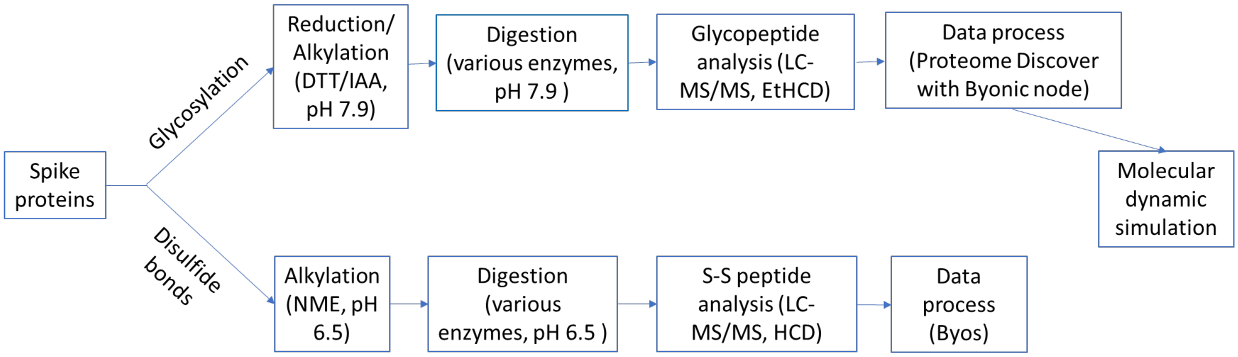

Figure S1. Workflow of the structural analysis of the SARS-CoV-2 Omicron and D614G spike proteins.

Table S1. N-Glycosylation analysis data of SARS-CoV-2 Omicron and D614G S proteins obtained from the experiments of various digestion conditions. The top row of each sequon was selected for discussion in the text of this report.

Sequon	S-Omicron						S-D614G						Digestion condition*
	Paucimannose	Oligomannose	Hybrid	Complex	Unoccupied	# Glycan	Paucimannose	Oligomannose	Hybrid	Complex	Unoccupied	# Glycan	
N17	14.6%	2.0%	3.0%	80.4%	0.0%	39	4.9%	0.7%	2.8%	91.6%	0.0%	34	LysC-LysC
	0.0%	0.0%	6.5%	93.5%	0.0%	11	0.0%	0.0%	6.1%	93.9%	0.0%	11	LysC-aLP
	0.0%	0.0%	4.6%	95.4%	0.0%	10	0.0%	0.0%	4.5%	95.5%	0.0%	10	LysC-CHTR
N61	7.0%	50.2%	19.8%	23.0%	0.0%	40	10.2%	62.6%	14.6%	12.6%	0.0%	38	LysC-aLP
	1.6%	51.3%	18.6%	28.5%	0.0%	32	2.0%	68.9%	14.0%	15.1%	0.0%	28	LysC-CHTR
	2.8%	54.3%	18.8%	24.1%	0.0%	35	4.5%	67.4%	14.3%	13.8%	0.0%	33	AspN-CHTR
N74	0.0%	0.1%	0.0%	99.9%	0.0%	37	0.3%	2.9%	3.8%	88.9%	4.0%	47	LysC-CHTR
	1.3%	22.6%	5.5%	63.8%	6.9%	30	0.0%	13.4%	0.6%	80.6%	5.4%	31	AspN-CHTR
N122	1.0%	61.4%	30.5%	7.1%	0.0%	43	1.5%	71.0%	22.2%	5.3%	0.0%	44	LysC-LysC
	0.8%	62.6%	30.6%	6.0%	0.0%	38	1.2%	71.0%	22.9%	4.9%	0.0%	35	LysC-Trypsin
	2.7%	52.3%	30.5%	14.5%	0.0%	32	2.6%	57.4%	20.5%	19.4%	0.0%	30	LysC-CHTR
N149	0.0%	37.4%	6.0%	56.6%	0.0%	17	0.0%	7.1%	11.7%	77.3%	3.9%	27	AspN-CHTR
	0.0%	20.3%	3.6%	65.8%	10.2%	16	0.0%	100.0%	0.0%	0.0%	0.0%	1	LysC-Trypsin
	0.0%	27.6%	4.7%	63.8%	3.9%	14	100.0%	0.0%	0.0%	0.0%	0.0%	1	LysC-LysC
	0.0%	66.1%	1.5%	30.2%	2.2%	24	0.0%	0.0%	0.0%	100.0%	0.0%	1	LysC-aLP
N165	0.1%	48.5%	46.9%	4.5%	0.0%	34	0.0%	35.0%	41.9%	23.1%	0.0%	43	LysC-Trypsin
	0.0%	35.5%	57.6%	6.8%	0.0%	32	0.1%	27.9%	51.8%	20.2%	0.0%	45	LysC-LysC
	1.5%	92.5%	4.4%	1.6%	0.0%	19	2.1%	73.7%	10.0%	14.3%	0.0%	29	LysC-CHTR
N234	0.4%	99.5%	0.0%	0.1%	0.0%	10	0.0%	99.4%	0.3%	0.3%	0.0%	12	LysC-CHTR
	0.0%	100.0%	0.0%	0.0%	0.0%	4	0.0%	100.0%	0.0%	0.0%	0.0%	6	LysC-aLP
	0.5%	92.0%	7.4%	0.0%	0.0%	12	0.3%	89.0%	10.5%	0.1%	0.0%	14	LysC-Trypsin
N282	0.6%	37.7%	27.8%	33.5%	0.4%	51	0.4%	27.0%	33.6%	38.6%	0.3%	52	LysC-LysC
	0.8%	33.5%	24.5%	40.9%	0.3%	51	0.7%	24.5%	34.4%	39.4%	0.9%	51	LysC-Trypsin
	0.0%	37.4%	27.2%	35.0%	0.4%	47	0.0%	27.6%	33.3%	38.7%	0.5%	46	LysC-CHTR
	0.0%	3.3%	34.4%	62.3%	0.0%	21	0.0%	0.0%	12.9%	87.1%	0.0%	19	LysC-aLP
N331	0.9%	21.1%	20.5%	57.5%	0.0%	40	0.7%	9.7%	23.0%	66.6%	0.0%	40	LysC-aLP
N343	1.7%	27.5%	21.8%	49.0%	0.0%	52	4.1%	28.1%	23.1%	44.6%	0.0%	52	LysC-aLP
N603	1.2%	98.4%	0.3%	0.0%	0.0%	13	1.4%	95.9%	2.7%	0.0%	0.0%	20	AspN-CHTR
	0.8%	94.8%	4.3%	0.1%	0.0%	9	0.9%	94.7%	3.3%	1.0%	0.0%	13	LysC-CHTR
N616	2.2%	47.1%	27.8%	22.9%	0.1%	44	2.7%	59.5%	28.5%	9.2%	0.1%	37	AspN-CHTR
	0.5%	51.2%	27.7%	20.6%	0.0%	31	0.8%	65.0%	28.6%	5.6%	0.0%	24	LysC-CHTR
N657	4.0%	23.2%	5.6%	46.4%	20.8%	30	1.4%	5.4%	3.7%	87.1%	2.4%	36	LysC-CHTR
	3.8%	17.8%	6.8%	39.4%	32.3%	32	0.6%	4.8%	5.1%	82.5%	7.0%	26	LysC-aLP
	0.1%	16.9%	4.1%	55.5%	23.4%	31	0.0%	7.8%	1.0%	78.7%	12.5%	13	LysC-Trypsin
N709	1.6%	97.9%	0.2%	0.3%	0.0%	10	2.2%	93.4%	1.6%	2.9%	0.0%	17	LysC-aLP
N717	1.3%	97.2%	1.5%	0.0%	0.0%	9	1.8%	95.7%	2.5%	0.0%	0.0%	10	LysC-aLP
N801	0.6%	99.1%	0.3%	0.0%	0.0%	12	1.3%	96.8%	0.8%	1.1%	0.1%	23	LysC-LysC
	1.5%	98.3%	0.2%	0.1%	0.0%	12	3.3%	94.9%	1.4%	0.3%	0.1%	19	LysC-aLP
	1.1%	97.9%	0.3%	0.5%	0.1%	12	2.5%	66.8%	1.0%	29.6%	0.1%	26	LysC-Trypsin
N1074	0.8%	87.9%	8.6%	2.0%	0.8%	42	0.7%	73.0%	17.6%	6.9%	1.8%	49	LysC-LysC
	0.9%	84.6%	10.7%	2.5%	1.3%	32	0.4%	77.4%	14.3%	6.1%	1.8%	37	LysC-Trypsin
	0.5%	88.9%	9.6%	0.4%	0.5%	21	1.1%	79.0%	14.2%	4.4%	1.3%	31	LysC-CHTR
	5.5%	82.4%	9.0%	3.1%	0.0%	12	6.6%	66.5%	16.4%	10.5%	0.0%	14	LysC-aLP
N1098	0.2%	46.5%	46.4%	6.1%	0.8%	43	0.3%	31.1%	57.6%	10.2%	0.8%	50	LysC-Trypsin
	0.4%	41.0%	48.4%	8.6%	1.6%	40	0.5%	24.2%	57.7%	16.0%	1.7%	42	LysC-CHTR
	0.0%	43.5%	54.2%	2.3%	0.0%	19	0.0%	27.6%	63.1%	9.3%	0.0%	23	LysC-LysC
	2.1%	37.4%	49.1%	8.3%	3.1%	39	1.9%	21.4%	58.6%	14.7%	3.4%	41	LysC-aLP
N1134	3.9%	18.5%	32.2%	44.9%	0.5%	72	3.3%	12.1%	40.9%	42.2%	1.4%	68	AspN-CHTR
	2.2%	2.4%	47.7%	47.8%	0.0%	18	2.9%	1.5%	51.6%	44.1%	0.0%	17	LysC-aLP
	0.0%	46.5%	25.7%	27.8%	0.0%	12	0.0%	37.1%	25.1%	37.8%	0.0%	12	LysC-Trypsin
	0.0%	35.3%	49.1%	15.5%	0.0%	14	0.0%	27.0%	49.0%	24.0%	0.0%	8	LysC-CHTR
	0.0%	28.4%	51.0%	20.6%	0.0%	7	0.0%	23.1%	57.3%	19.7%	0.0%	7	LysC-LysC
N1158	1.6%	9.2%	6.9%	81.9%	0.4%	30	0.7%	2.3%	3.6%	92.6%	0.8%	30	LysC-aLP
	0.5%	8.5%	9.6%	81.4%	0.0%	27	0.0%	1.2%	0.1%	98.7%	0.0%	31	AspN-CHTR
N1173	0.8%	7.6%	3.0%	47.6%	41.0%	46	0.2%	1.0%	0.9%	40.6%	57.3%	44	AspN-CHTR
	0.4%	5.2%	5.6%	88.9%	0.0%	32	0.2%	1.1%	2.2%	96.5%	0.0%	32	LysC-aLP
N1194	0.0%	5.6%	2.0%	56.3%	36.0%	45	0.0%	1.0%	0.6%	46.1%	52.3%	40	LysC-LysC
	0.5%	6.2%	3.2%	57.3%	32.8%	45	0.0%	1.0%	0.8%	39.8%	58.4%	37	LysC-Trypsin

* The proteins were sequentially digested by listed combination of two enzymes with the first digestion conducted at 52°C for 60 min and the second one at 37°C overnight. CHTR and aLP represent chymotrypsin and α-lytic protease, respectively.

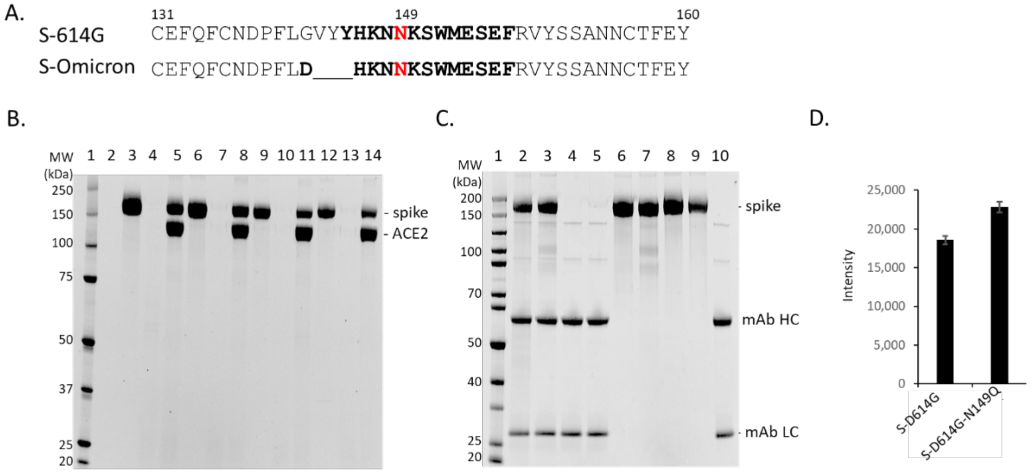

Figure S2. (**A**) The region of the S-D614G and S-Omicron sequences containing the N149 sequon. Detected glycopeptides from these two proteins are displayed in bold. (**B**) SDS-PAGE analysis of the spike proteins binding to ACE2. Lane 1: marker; 2: empty; 3: S-D614G control in solution; 4: S-D614G and protein G beads; 5: S-D614G and ACE2 immobilized on protein G beads; 6: S-Omicron; 7: S-Omicron and protein G beads; 8: S-Omicron and ACE2 on protein G beads; 9: S-D614G-N149Q; 10: S-D614G-N149Q and protein G; 11: S-D614G-N149Q and ACE2-protein G beads; 12: S-Omicron-N149Q; 13: S-Omicron-N149Q and protein G; 14: S-Omicron-N149Q and ACE2-protein G beads. (**C**) SDS-PAGE analysis of 4A8 mAb binding to the spike proteins. Lane1: marker; 2: S-D614G and mAb-protein G beads; 3: S-D614G-N149Q and mAb-protein G beads; 4: S-Omicron and mAb-protein G beads; 5: S-Omicron-N149Q and mAb-protein G beads; 6: S-D614G control in solution; 7: S-D614G-N149Q; 8: S-Omicron; 9: S-Omicron-N149Q; 10: mAb-protein beads control. (D) The intensities of the S-D614G and S-D614G-N149Q bands binding to 4A8 antibody. Densitometry values represent the average of three independent experiments and the error bars indicate the standard deviation around the average. One microgram of S, ACE2 and mAb material was used for all experiments.

Table S2. Peak areas of the disulfide bonded (DB) peptides detected in both SARS-CoV-2 spike proteins of D614G and Omicron variants digested by various proteases.

Digestion protease(s)[a]	DB #[b]	Peptide 1[c]		Peptide 2		D614G		Omicron		Ratio (Omicron/ D614G)
		Sequence	C1 posit.	Sequence	C2 posit.	Avg	SD	Avg	SD	
E3	DB3	DAVDCA	291	CTLK	301	1.4E+09	4.0E+08	1.7E+09	6.4E+08	123%
E4	DB3	DAVDCAL	291	CTLK	301	1.7E+09	4.2E+08	1.7E+09	2.6E+08	98%
E4	DB3	DAVDCALDPLSETK	291	CTLK	301	8.1E+08	7.2E+07	5.0E+08	1.4E+08	62%
E4	DB3	DCAL	291	CTLK	301	5.8E+08	2.5E+08	9.1E+08	1.7E+08	157%
E3	DB3	DCALDPLSET	291	CTLK	301	9.7E+07	1.8E+07	1.5E+08	3.6E+07	158%
E3	DB3	DCALDPLSETK	291	CTLK	301	9.3E+08	7.3E+07	1.0E+09	1.5E+08	109%
E4	DB3	DCALDPLSETK	291	CTLK	301	6.6E+08	1.7E+08	4.1E+08	4.4E+07	62%
E2	DB3	YNENGTITDAVDCALDPLSETKCTLK	291		301	9.4E+06	2.5E+05	1.0E+07	2.7E+06	109%
E1	DB4	CPF	336	ISNCVADY	361	1.2E+08	1.5E+07	3.9E+07	1.4E+07	32%
E1	DB4	CPFGEVF	336	ISNCVADY	361	6.9E+07	1.9E+07	1.6E+07	4.2E+06	23%
E3	DB4	NLCPFG(D)EV	336	NCVA	361	2.3E+08	1.8E+07	3.0E+08	6.3E+07	131%
E1	DB5	CY	379	KLPDDFTGCVIAW	432	4.9E+08	1.8E+08	2.8E+08	7.2E+07	56%
E3	DB5	CYGVSPT	379	GCVIA	432	3.4E+09	3.6E+08	2.3E+09	4.4E+08	68%
E1	DB5	KCY	379	LPDDFTGCVIAW	432	5.0E+08	1.9E+08	2.3E+08	7.7E+07	46%
E1	DB5	KCY	379	KLPDDFTGCVIAW	432	1.8E+09	1.8E+08	4.5E+08	7.8E+07	25%
E1	DB6	CF	391	HAPATVCGPK	525	2.7E+08	2.5E+07	1.0E+08	3.6E+07	37%
E3	DB6	KLNDLCFT	391	TVCGPK	525	1.6E+09	2.8E+08	1.7E+09	6.4E+08	105%
E3	DB6	KLNDLCFT	391	TVCGPKK	525	2.7E+09	4.6E+08	1.9E+09	1.1E+08	71%
E3	DB6	KLNDLCFT	391	CGPK	525	5.8E+08	1.2E+08	4.7E+08	4.9E+07	81%
E3	DB6	KLNDLCFT	391	CGPKK	525	3.7E+08	7.0E+07	2.3E+08	2.7E+07	62%
E1	DB6	LNDLCF	391	HAPATVCGPKK	525	2.7E+09	2.8E+08	1.1E+09	2.6E+08	43%
E3	DB6	LNDLCFT	391	TVCGPK	525	6.4E+08	8.8E+07	8.7E+08	1.2E+08	137%
E3	DB6	LNDLCFT	391	TVCGPKK	525	1.1E+09	1.8E+08	1.3E+09	1.6E+08	122%
E3	DB6	LNDLCFT	391	CGPK	525	2.7E+08	4.2E+07	3.5E+08	5.8E+07	130%
E3	DB6	LNDLCFT	391	CGPKK	525	2.4E+08	1.8E+07	2.7E+08	4.9E+07	111%
E2	DB6	LNDLCFTNVYADSFVIR	391	VVVLSFELLHAPATVCGPK	525	1.3E+08	1.4E+07	1.1E+08	3.9E+07	83%
E1	DB6	NDLCF	391	HAPATVCGPK	525	9.8E+08	1.1E+08	3.7E+08	6.5E+07	38%
E1	DB6	NDLCF	391	HAPATVCGPKK	525	1.0E+08	2.1E+07	5.0E+07	1.3E+07	49%
E3	DB8	CV	538	PCSFGGV	590	5.3E+08	3.5E+07	3.7E+08	7.4E+07	71%
E3	DB8	CV	538	PCSFGGVS	590	8.2E+08	1.1E+08	5.5E+08	1.1E+08	67%
E1	DB8	CVNF	538	DITPCSF	590	2.3E+09	8.1E+08	3.7E+09	1.3E+09	162%
E1	DB8	CVNF	538	EILDITPCSF	590	1.5E+09	7.5E+08	3.4E+09	6.2E+08	218%
E3	DB8	CVNFNFNGLT(K)	538	PCSFGGV	590	1.4E+09	3.7E+08	1.1E+09	1.1E+08	82%
E1	DB8	NKCVNF	538	DITPCSF	590	1.8E+09	3.9E+08	1.5E+09	2.3E+08	80%
E1	DB8	NKCVNF	538	EILDITPCSF	590	1.3E+09	2.9E+08	1.0E+09	2.4E+08	79%
E3	DB10	NNSYECDIPIGAGICAS	662		671	1.9E+08	2.9E+07	8.6E+08	8.8E+07	439%
E3	DB11	TSVDCT	738	FCT	760	1.9E+08	4.1E+07	1.4E+08	2.9E+07	75%
E1	DB11	TSVDCTMY	738	CTQL	760	2.2E+09	1.6E+08	2.6E+09	4.8E+08	119%
E1	DB11	TSVDCTMY	738	GSFCTQL	760	3.3E+09	5.1E+08	4.7E+09	1.4E+09	142%
E1	DB12	ICGDSTECSNL	743		749	4.8E+09	4.6E+08	6.6E+09	1.7E+09	138%
E1	DB12	ICGDSTECSNLL	743		749	3.8E+08	4.3E+07	3.2E+08	3.4E+07	84%
E3	DB12	MYICGDSTECS	743		749	7.5E+08	3.6E+07	7.9E+08	4.9E+07	105%
E4	DB13	DCLG	840	DLICAQK	851	7.8E+09	1.8E+09	7.6E+09	5.4E+08	98%
E1	DB13	GDCLGDIAAR	840	DLICAQK	851	4.6E+08	1.5E+08	1.7E+09	4.6E+08	365%
E1	DB13	GDCLGDIAAR	840	DLICAQKF	851	1.3E+09	3.4E+08	9.0E+08	1.6E+08	68%
E4	DB13	QYGDCLG	840	DLICAQK	851	4.9E+08	1.9E+08	5.6E+08	9.8E+07	114%
E3	DB13	QYGDCLGDIAA	840	RDLICAQK	851	2.1E+08	1.6E+07	3.4E+08	1.0E+08	162%
E2	DB13	QYGDCLGDIAAR	840	DLICAQK	851	1.0E+10	1.3E+09	1.4E+10	4.2E+09	140%
E1	DB13	QYGDCLGDIAAR	840	DLICAQKF	851	4.1E+08	1.7E+08	5.1E+08	7.8E+07	124%
E3	DB13	QYGDCLGDIAARDLICAQK	840		851	1.4E+10	2.4E+09	1.5E+10	3.4E+09	108%
E3	DB14	MSECV	1032	RVDFCGK	1043	8.6E+08	5.2E+07	5.1E+08	9.5E+07	59%
E1	DB14	MSECVL	1032	CGK	1043	2.0E+08	1.8E+07	1.1E+08	2.0E+07	59%
E1	DB14	MSECVL	1032	RVDFCGK	1043	1.6E+09	3.0E+08	3.2E+09	6.0E+08	207%
E1	DB14	MSECVLGQSK	1032	RVDFCGK	1043	2.8E+08	6.2E+07	3.0E+08	7.1E+07	111%
E2	DB14	MSECVLGQSK	1032	RVDFCGK	1043	5.8E+09	3.6E+08	6.8E+09	6.1E+08	116%
E4	DB14	MSECVLGQSK	1032	RVDFCGK	1043	6.5E+09	1.7E+09	4.2E+09	2.6E+08	65%
E2	DB14	MSECVLGQSK	1032	VDFCGK	1043	5.0E+08	1.3E+08	5.2E+08	7.7E+06	105%
E4	DB14	MSECVLGQSK	1032	VDFCGK	1043	2.0E+09	3.1E+08	1.8E+09	8.4E+07	91%
E2	DB14	MSECVLGQSKR	1032	VDFCGK	1043	4.8E+09	2.3E+08	5.6E+09	6.5E+08	117%
E4	DB14	MSECVLGQSKR	1032	VDFCGK	1043	5.3E+09	1.2E+09	5.1E+09	2.1E+09	96%
E2	DB14	MSECVLGQSKRVDFCGK	1032		1043	2.3E+08	2.5E+07	2.6E+08	2.0E+07	115%
E3	DB15	ICHDGK	1082	GNCDVV	1126	2.1E+09	4.6E+08	1.9E+09	2.4E+08	91%
E3	DB15	ICHDGK	1082	SGNCDVV	1126	1.5E+09	3.9E+08	1.1E+09	2.8E+08	75%
E3	DB15	ICHDGKA	1082	GNCDVV	1126	3.0E+08	5.7E+07	2.0E+08	2.8E+07	66%
E3	DB15	ICHDGKA	1082	SGNCDVV	1126	1.3E+08	3.3E+07	1.3E+08	1.0E+07	100%
E4	DB15	NFTTAPAICH	1082	DNTFVSGNC	1126	9.6E+07	1.1E+07	3.4E+07	3.8E+06	36%

a. enzyme(s): E1, E2, E3, and E4 represent trysin + chymotrypsin, Lys-C + trypsin, Lys-C + alphalitic protease, and Asp-N + trypsin, respectively.

b. The peptides contains cystine residue but do not form a disulfide bond are included. For example, DB3-free C1 represent a peptide with NEM modified free Cys at 391 position 291.

c. The sequence and the amino acid position of the peptides are derived from the D614G spike. Letters in parentheses represent substitutions of Omicron spike protein.

A

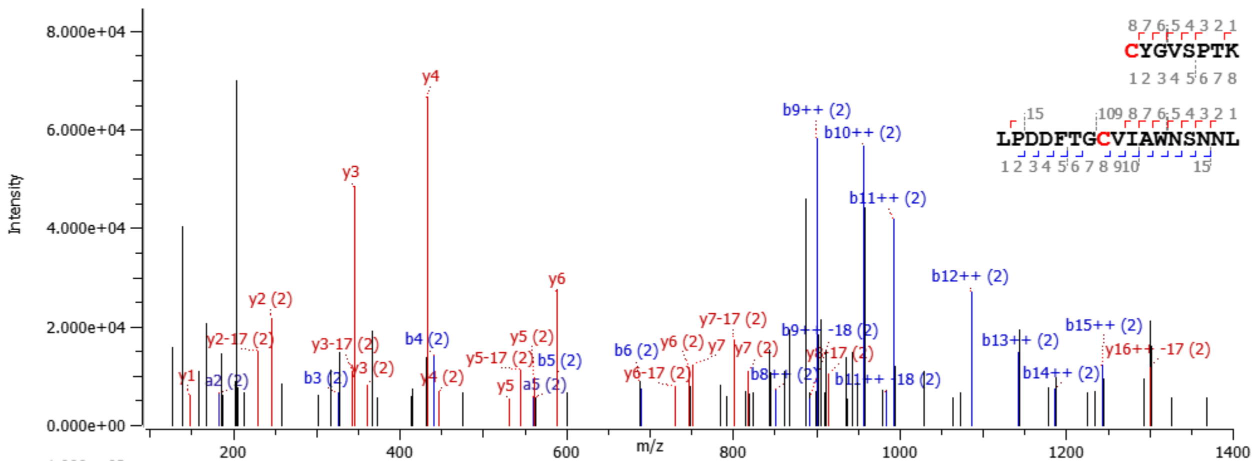

B

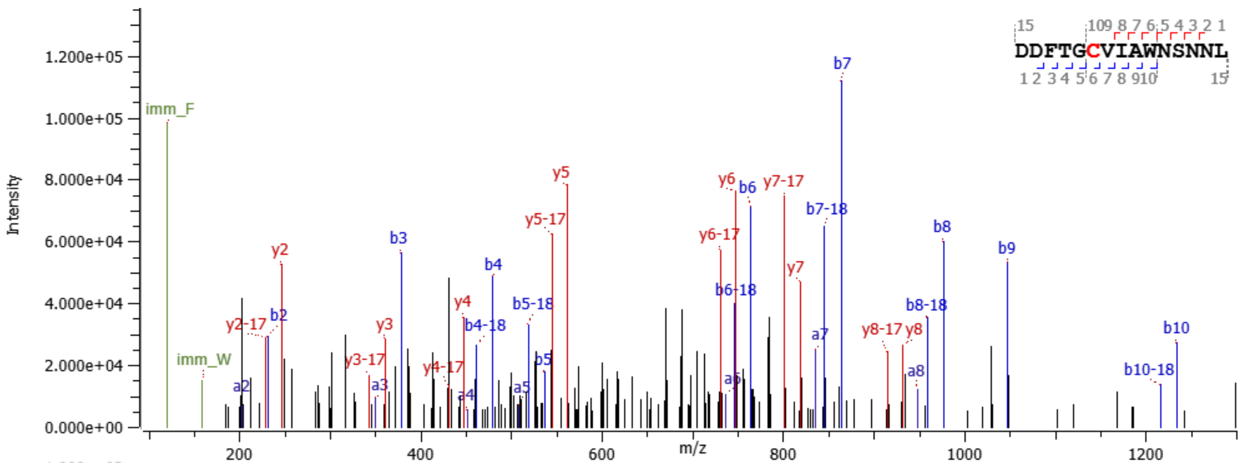

Figure S3. MS/MS spectra of the C379-C432 peptide (**A**) and the peptide bearing the free C432 cysteine residue modified by NEM (**B**).

Table S3. Glycan constituents at various positions in the 614G and Omicron S proteins.

Site	S-D614G	S-Omicron
N17	N5H4A1	N2H4F1
N61	N2H5	N2H5
N74	N4H3F1	N4H5F1A1
N122	N2H5	N2H5
N149	N4H5F1A1	N2H5
N165	N3H4	N3H4
N234	N2H9	N2H9
N282	N2H5	N2H5
N331	N4H5F1A1	N4H4F1
N343	N2H5	N2H5

N603	N2H5	N2H5
N616	N2H5	N2H5
N657	N4H4F1	N2H5
N709	N2H7	N2H8
N717	N2H7	N2H7
N801	N2H7	N2H8
N1074	N2H5	N2H5
N1098	N3H5	N2H5
N1134	N3H4F1	N3H4F1
N1158	N5H3F1	N5H3F1

* N, H, F, and A represent N-acetyl hexosamine (HexNac), hexose (Hex), fucose (Fuc), and N-acetylneuraminic acid (NeuAc) groups, respectively, in glycan compositions.

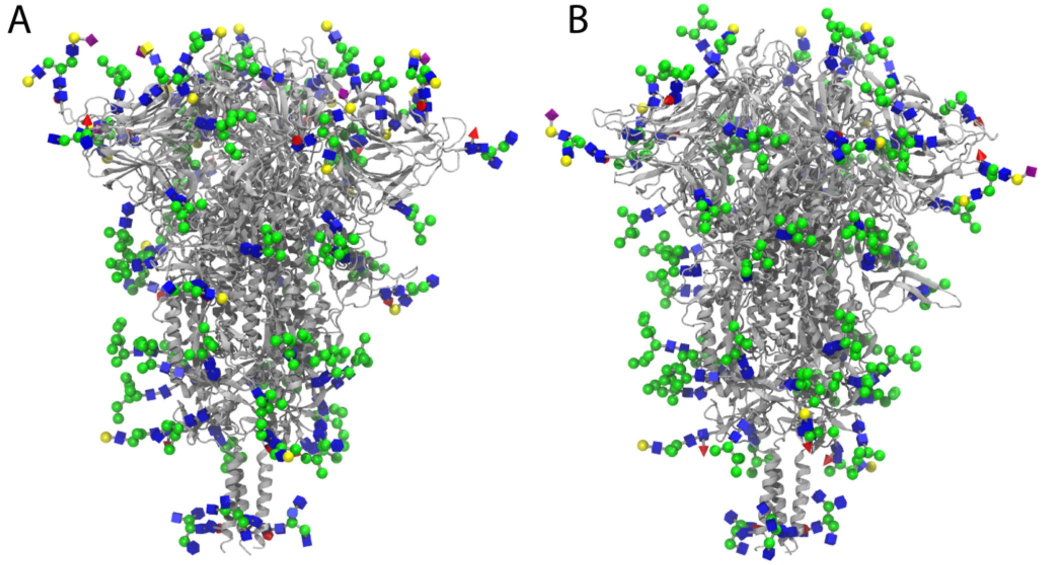

Figure S4. Constructed glycosylated SARS-CoV-2 spike proteins of D614G (A) and Omicron (B) variants. The attached glycans are visualized using 3D-SNFG (symbol nomenclature for glycans) representations[2].

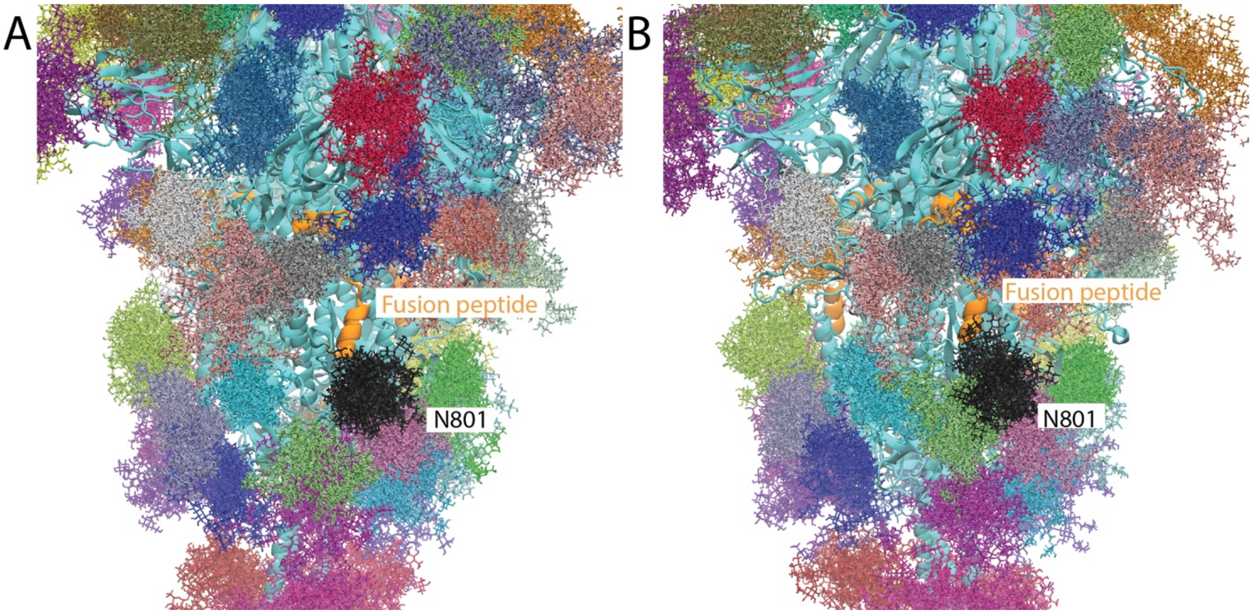

Figure S5. Glycan shielding near the fusion peptide. The D614G (**A**) and Omicron (**B**) S proteins are depicted in cyan using a cartoon representation. The superimposed glycans are represented by colorful licorice models. These glycan configurations were captured at intervals of 0.25 μs throughout the net 4.2 μs of simulation trajectories for the D614G and Omicron S proteins. Fusion peptides are highlighted in orange.

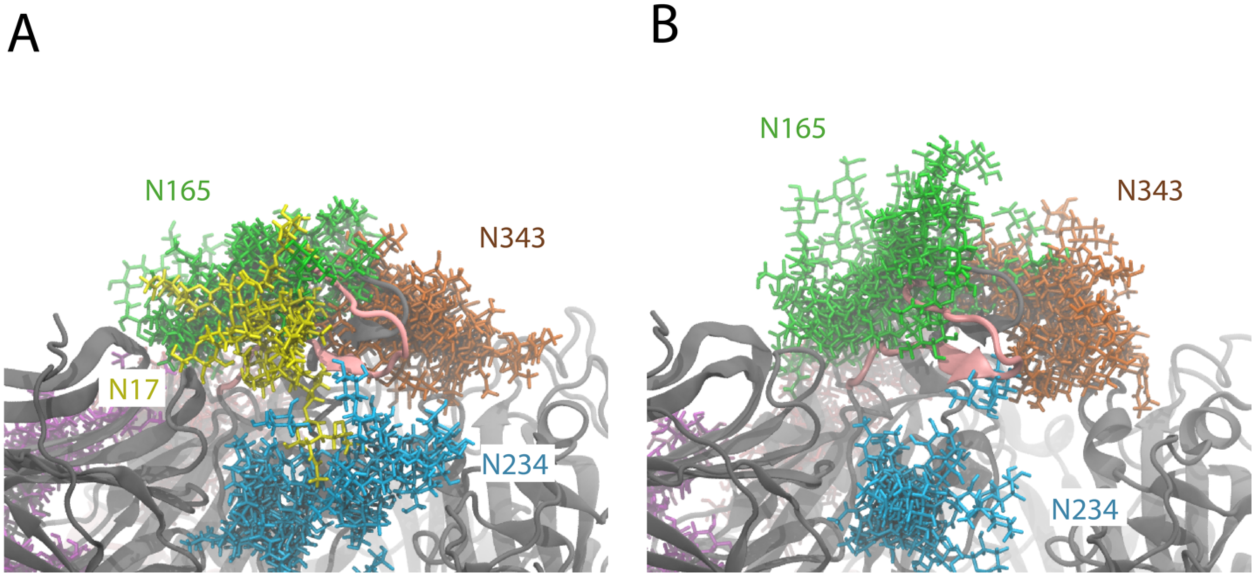

Figure S6. Glycan shielding of the receptor binding domain (RBD) in the SARS-CoV-2 S protein. The residues from S469 to V483 of chain A in the D614G (**A**) and Omicron (**B**) S proteins are shown in pink. All glycan residues within 5 Å of the RBDs are depicted in the figures. These structures are superimposed at intervals of 0.25 μs along the respective simulation trajectories.

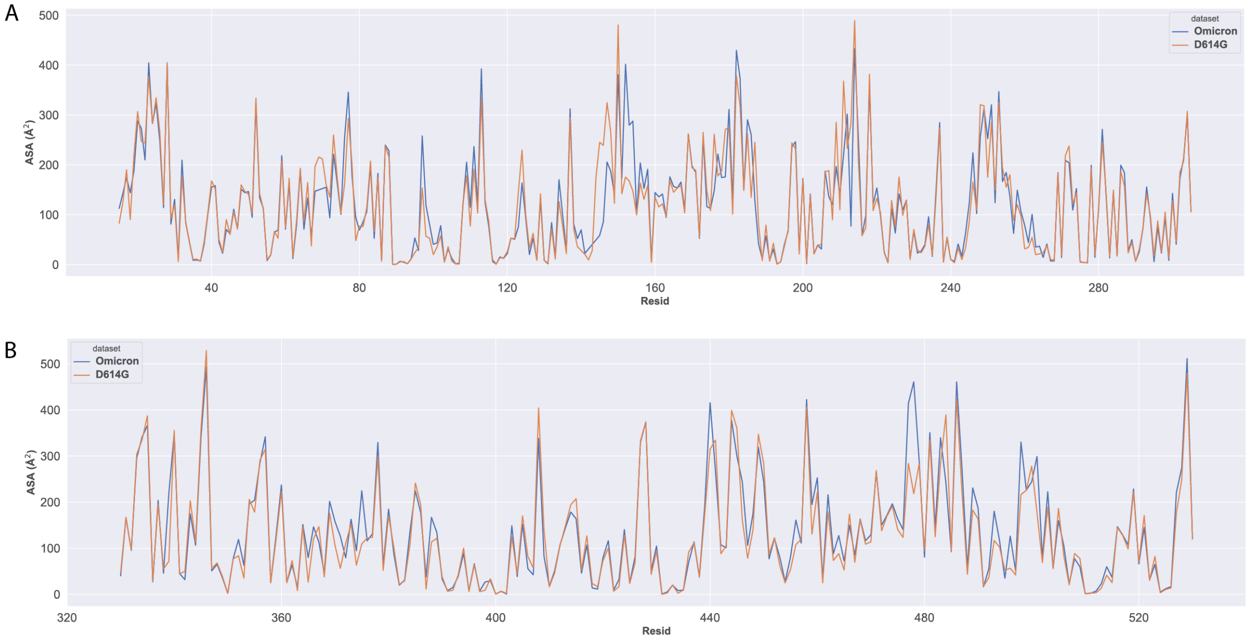

Figure S7. The accessible surface area (ASA) of the N-terminal domain (A) and receptor binding domain (B), factoring in the presence of glycans. The ASA is measured in $Å^2$.

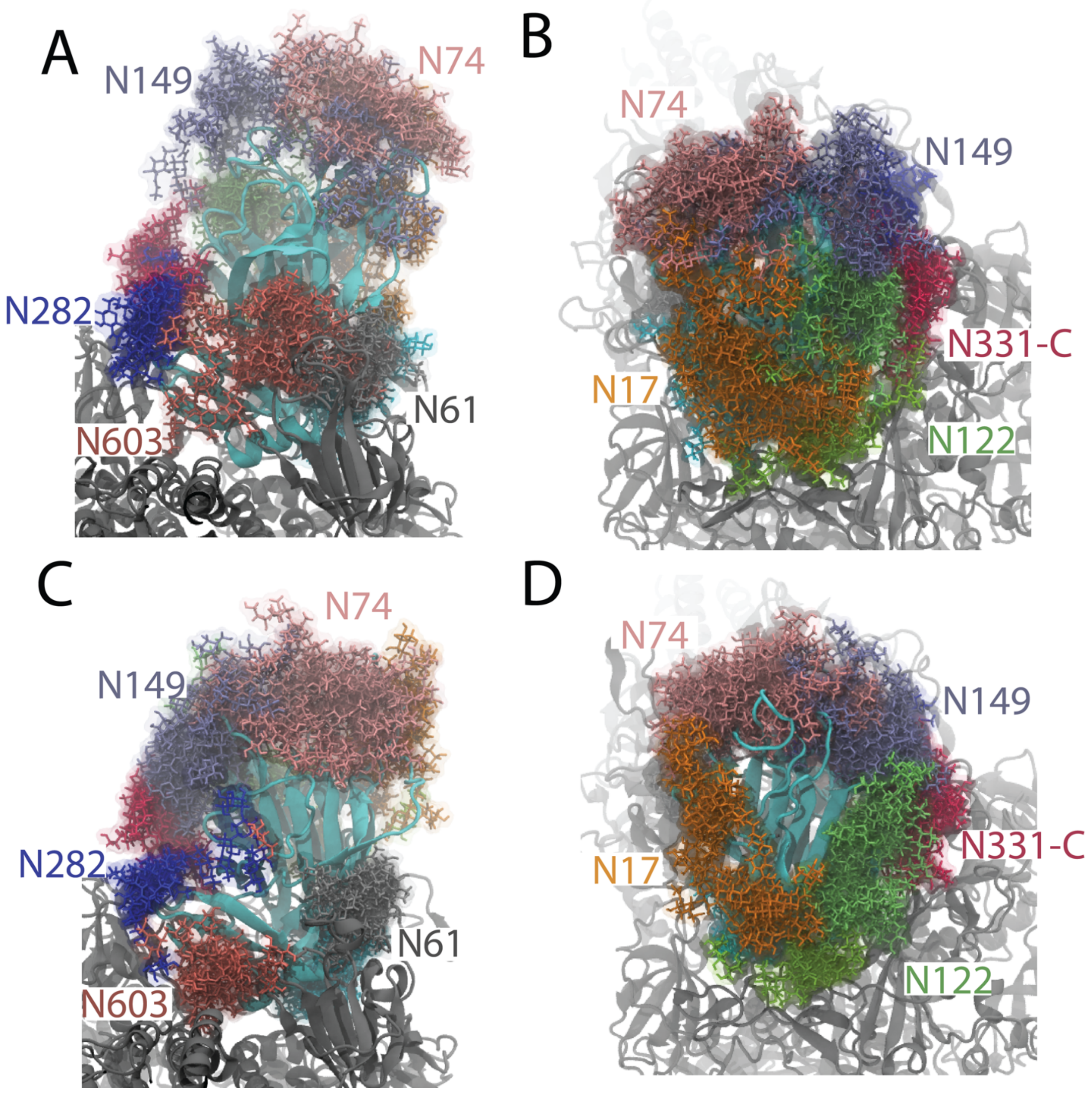

Figure S8. Glycan shielding of the N-terminal domain (NTD) in the SARS-CoV-2 S protein. The NTDs of chain A in the D614G (**A, B**) and Omicron (**C, D**) S proteins are shown in cyan. All glycan residues within 5 Å of the NTDs are depicted in the figures. These structures are superimposed at intervals of 0.25 μs along the respective simulation trajectories.

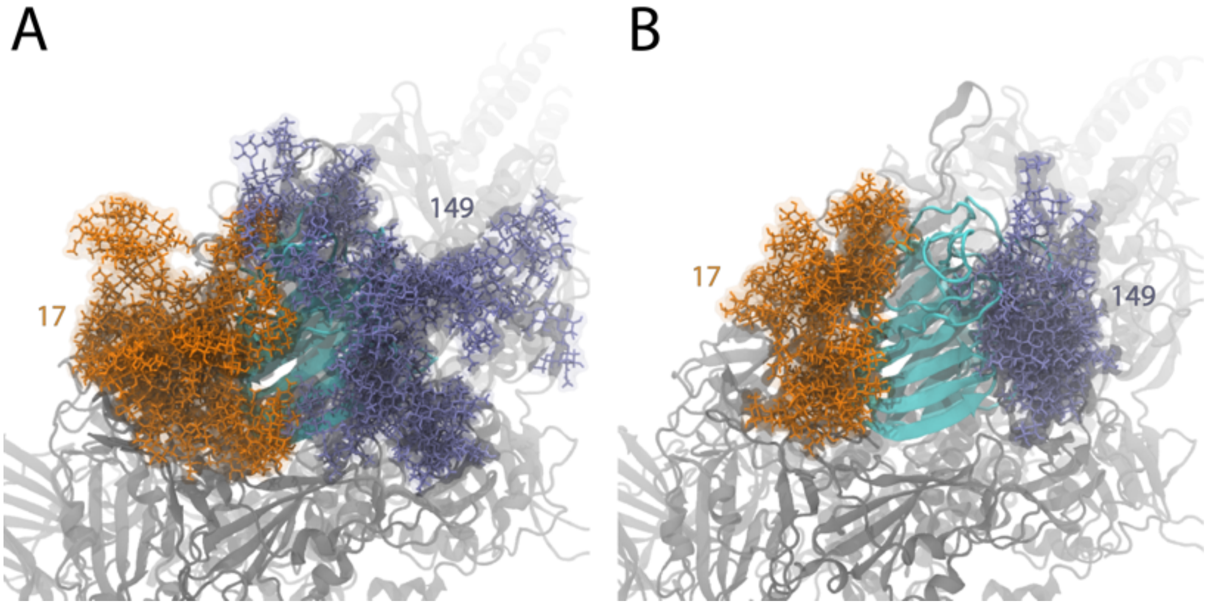

Figure S9. Full-length glycan coverage at positions N17 and N149. Glycans are depicted in their complete length. The displayed structures are cumulative snapshots taken at 0.25-µs intervals along the respective simulation trajectories.

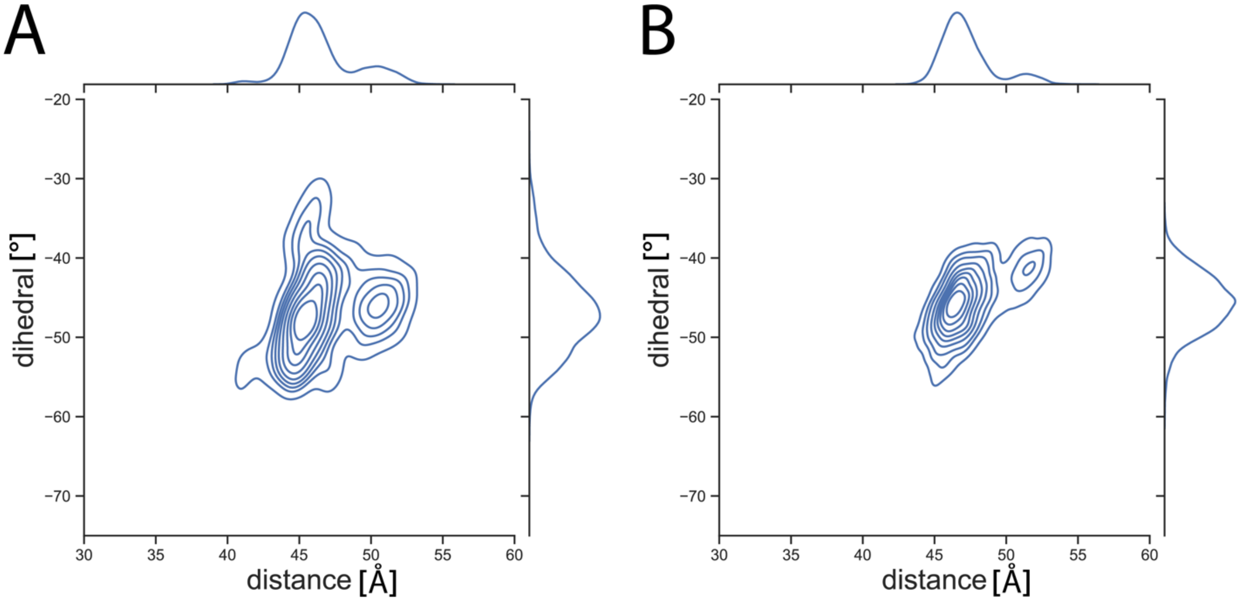

Figure S10. RBD conformation in the D614G S protein. Two-dimensional kernel density estimate (KDE) plot visualizes the spread of RBD conformations within the trajectories of the un-glycosylated (A) and glycosylated (B) systems, with two collective variables (defined in Methods), a distance and a dihedral angle, characterizing the RBD conformations.

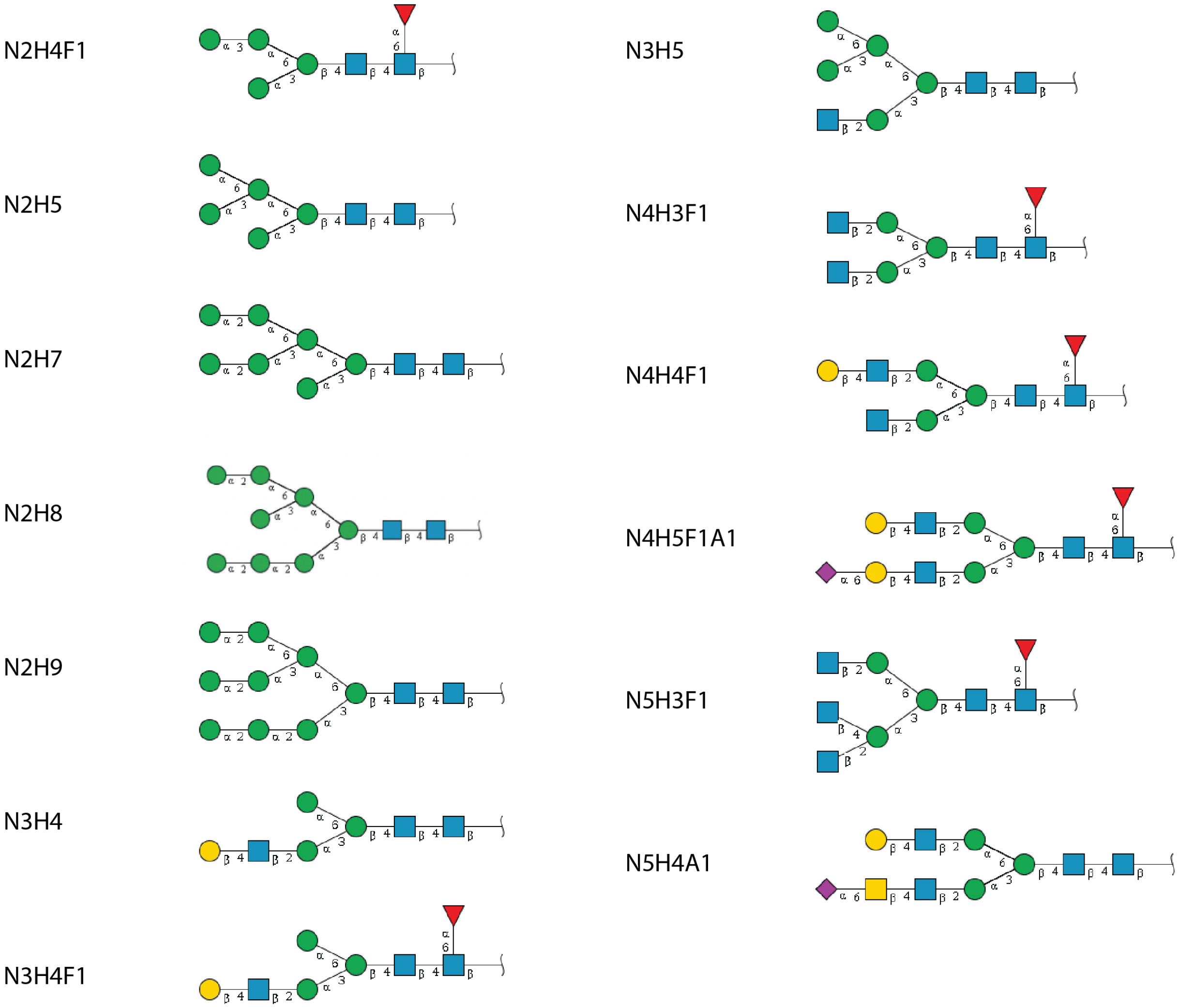

Figure S11. Specific glycan structures for various glycan constituents generated from the website GlyGen website (https://www.glygen.org)[1].

Reference

1. York, W. S.; Mazumder, R.; Ranzinger, R.; Edwards, N.; Kahsay, R.; Aoki-Kinoshita, K. F.; Campbell, M. P.; Cummings, R. D.; Feizi, T.; Martin, M.; Natale, D. A.; Packer, N. H.; Woods, R. J.; Agarwal, G.; Arpinar, S.; Bhat, S.; Blake, J.; Castro, L. J. G.; Fochtman, B.; Gildersleeve, J., GlyGen: Computational and Informatics Resources for Glycoscience. *Glycobiology.* **2020,** *30* (2), 72-73.
2. Thieker, D. F.; Hadden, J. A.; Schulten, K.; Woods, R. J., 3D implementation of the symbol nomenclature for graphical representation of glycans. *Glycobiology.* **2016,** *26* (8), 786-787.
